# The dose-response relationship between socioeconomic deprivation and alcohol-attributable mortality risk—a systematic review and meta-analysis

**DOI:** 10.1186/s12916-021-02132-z

**Published:** 2021-11-05

**Authors:** Charlotte Probst, Shannon Lange, Carolin Kilian, Celine Saul, Jürgen Rehm

**Affiliations:** 1 Institute for Mental Health Policy Research, Centre for Addiction and Mental Health (CAMH), Toronto, ON M5S 2S1 Canada; 2 Heidelberg Institute of Global Health (HIGH), Medical Faculty and University Hospital, Heidelberg University, 69120 Heidelberg, Germany; 3 Campbell Family Mental Health Research Institute, Centre for Addiction and Mental Health, Toronto, ON M5S 2S1 Canada; 4 Department of Psychiatry, University of Toronto, Toronto, ON M5T 1R8 Canada; 5 Institute of Clinical Psychology and Psychotherapy, Technische Universität Dresden, 01187 Dresden, Germany; 6 Dalla Lana School of Public Health, University of Toronto, M5T 3 M7, Toronto, ON Canada; 7 Centre for Interdisciplinary Addiction Research, University of Hamburg, 20246 Hamburg, Germany; 8 Department of International Health Projects, Institute for Leadership and Health Management, I.M. Sechenov First Moscow State Medical University, Moscow, Russian Federation 125009

**Keywords:** Socioeconomic status, Inequality, Dose-response, Socioeconomic deprivation, Alcohol use, Mortality, Public health

## Abstract

**Background:**

Individuals with low socioeconomic status (SES) experience a higher risk of mortality, in general, and alcohol-attributable mortality in particular. However, a knowledge gap exists concerning the dose-response relationships between the level of socioeconomic deprivation and the alcohol-attributable mortality risk.

**Methods:**

We conducted a systematic literature search in August of 2020 to update a previous systematic review that included studies published up until February of 2013. Quantitative studies reporting on socioeconomic inequality in alcohol-attributable mortality among the general adult population were included. We used random-effects dose-response meta-analyses to investigate the relationship between the level of socioeconomic deprivation and the relative alcohol-attributable risk (RR), by sex and indicator of SES (education, income, and occupation).

**Results:**

We identified 25 eligible studies, comprising about 241 million women and 230 million men, among whom there were about 75,200 and 308,400 alcohol-attributable deaths, respectively. A dose-response relationship between the level of socioeconomic deprivation and the RR was found for all indicators of SES. The sharpest and non-linear increase in the RR of dying from an alcohol-attributable cause of death with increasing levels of socioeconomic deprivation was observed for education, where, compared to the most educated individuals, individuals at percentiles with decreasing education had the following RR of dying: women: 25th: 2.09 [95% CI 1.70–2.59], 50th: 3.43 [2.67–4.49], 75th: 4.43 [3.62–5.50], 100th: 4.50 [3.26–6.40]; men: 25th: 2.34 [1.98–2.76], 50th: 4.22 [3.38–5.24], 75th: 5.87 [4.75–7.10], 100th: 6.28 [4.89–8.07].

**Conclusions:**

The findings of this study show that individuals along the entire continuum of SES are exposed to increased alcohol-attributable mortality risk. Differences in the dose-response relationship can guide priorities in targeting public health initiatives.

**Supplementary Information:**

The online version contains supplementary material available at 10.1186/s12916-021-02132-z.

## Background

Low socioeconomic status (SES) has repeatedly been shown to be associated with an elevated risk of mortality [1–3]. A study that included data from 15 European countries found that compared to their high SES counterparts, men with low SES (measured through education) had a lowered life expectancy at age 35 of 2 to 8 years and women with low SES lost from 0.6 to nearly 5 years of life expectancy at age 35, depending on the country [4]. A large multicohort study including more than 1.7 million participants investigated the impact of low SES (measured through occupation) and six major behavioral risk factors (high alcohol use, physical inactivity, current smoking, hypertension, diabetes, and obesity) on life expectancy at age 40 [5]. The authors found that, while adjusting for all the risk factors under consideration, when compared to high SES, low SES was associated with a reduction in life expectancy by more than 2 years.

Socioeconomic inequalities are particularly wide for alcohol-attributable causes of death. A systematic review and meta-analysis found that the relative socioeconomic inequalities in mortality were about 1.5 to two times higher for alcohol-attributable mortality, compared to socioeconomic inequalities in all-cause mortality [6]. While alcohol use itself was found to explain less than 30% of the socioeconomic inequalities in all-cause and alcohol-attributable mortality, there is some evidence of joint effects between alcohol use and low SES, contributing to the increased socioeconomic inequality in alcohol-attributable mortality [7, 8].

The majority of previous overview papers and meta-analyses on socioeconomic inequalities in alcohol-attributable mortality have focused on the extreme-group comparison between the lowest and the highest SES group, neglecting the other SES groups in between which are crucial for understanding the dose-response relationship between SES and relative mortality risks [9–11]. The current meta-analysis addresses this research gap by considering all pairwise comparisons and translating them into a continuous framework. Furthermore, previous overview works were not able to compare different indicators of SES with regard to the strengths of their association with the alcohol-attributable mortality risk as they were not able to separate “true” differences between indicators of SES that arise from their individual causal pathways [12] from the differences that arise from conventional levels of grouping that tie the indicator of SES to the extent to which an extreme group comparison is being reported (e.g., for income, quintiles are conventionally used, leading to a comparison between the lowest and the upper 20% of the SES distribution, whereas for education three broad categories are more common). Due to this inter-dependence, previous meta-analyses were unable to interpret the observed differences in socioeconomic inequalities conditional on the indicator of SES used [9]. Thus, to date, the current meta-analysis is the first to systematically investigate the differences between indicators of SES, independent of the conventional groupings used for different indicators of SES. Furthermore, there is some evidence for sex differences in the socioeconomic inequality of alcohol-attributable mortality, with men having a higher relative risk (RR) [9]. However, besides occupation, existing meta-analyses did not have sufficient statistical power (i.e., did not have sufficient studies available; in practice, five or more studies are necessary to achieve sufficient power in a random-effects meta-analysis compared to the individual studies contributing to it [13]) to demonstrate sex differences for other indicators of SES.

Accordingly, the present study pursued the following two objectives:

1. Investigate differences in the strength of the association between different indicators of SES (education, income, and occupation) and the alcohol-attributable mortality risk, by sex; and
2. Investigate the sex-specific dose-response relationship between the level of socioeconomic deprivation and the relative alcohol-attributable mortality risk for core indicators of SES, by sex.

## Methods

The study protocol of the present systematic review and meta-analysis followed the Preferred Reporting Items for Systematic Reviews and Meta-Analyses (PRISMA [14]; Additional file 1: Table S1) and was preregistered in PROSPERO (registration number CRD42019140279).

### Systematic literature search

A systematic literature search was conducted during the last week of August 2020 in Embase, MEDLINE, PsycINFO, and Web of Science to update a previous systematic review performed by our group that included all studies published from journal inception to the second week of February 2013 [6, 9]. Search terms relating to alcohol consumption, mortality, SES, and study design were used and adapted to each of the databases searched to include truncations and medical subject headings where applicable (Additional file 1: Text S1). No language or geographical restrictions were applied. References and citing articles of identified studies were manually screened.

### Study selection and inclusion criteria

Studies were included if they (i) consisted of original, quantitative research; (ii) reported the relative alcohol-attributable mortality risk by education, income, or occupation as the indicator of SES, including a measure of uncertainty or sufficient original data to calculate the risk and/or uncertainty; (iii) provided the proportion of individuals in each SES category; and (iv) were based on a sample from the general adult (15+ years) population. Studies that involved specific sub-populations (e.g., clinical samples) were excluded. Studies that used a longitudinal, cross-sectional, or case-control design were eligible. Studies that did not report results by sex were eligible to be included in the qualitative synthesis only. For detailed inclusion and exclusion criteria, see Additional file 1: Table S2. Alcohol-attributable causes of death were defined as all causes of death that are fully attributable to alcohol use [15] or causes of death with an alcohol-attributable fraction of 10% or more globally (see Additional file 1: Tables S3 and S4 for ICD-10 codes) [16].

Articles were screened by three reviewers, first using titles and abstracts, then full texts for those identified as potentially eligible. When eligibility was unclear, inclusion was decided by consensus following a discussion of the respective study. To reach a high agreement (Kappa> 0.8) [17], a subsample of 50 references was used to train reviewers. Unless they reported results for different indicators of SES, studies with overlapping data sources were excluded to prevent double-counting of observations. Study quality (see below) was used to guide decisions regarding study and estimate inclusion in cases of overlapping data.

### Data extraction

The following information was extracted: study population, study design, mortality assessment, ICD-10 codes, assessment of SES, sample size, death counts by SES, proportions of the sample in each SES group, results, and adjustment for confounding. For the results, all pairwise comparisons reported per study were included to facilitate dose-response analyses. Data were extracted by three reviewers; disagreements were resolved via consensus-based discussion. Hazard ratios, RRs, and mortality rate ratios were treated as equivalent measures of relative mortality risk. Age-adjusted and sex-stratified estimates were preferentially extracted. Data extracted for the review performed in 2013 were re-checked and merged with the newly extracted data [18].

### Coding of the level of socioeconomic deprivation

In order to investigate the dose-response relationship between the level of socioeconomic deprivation and the mortality risk, the single pairwise comparisons reported in each study had to be converted to a unified and continuous scale. For this purpose, we used percentiles in the cumulative SES distribution to convert the *k* groups of SES used in each study for pairwise comparisons to a continuous or level of socioeconomic deprivation (the “dose”). For each of the *k* SES groups the fraction *f*_*i*_ of the total sample in group *i* was calculated. Then, the groups were ordered from the lowest level of socioeconomic deprivation (that is the group with the highest SES) to the highest level of deprivation (*i* = 1, …, *i* = *k*), adding up to a 100% in total ($\sum \limits_{i=1}^k{f}_i=100\%$). With this, we determined the percentile range *PR* for each SES group (${PR}_i=\left[\sum \limits_1^{i-1}{f}_i,\sum \limits_{i=1}^k{f}_i\right]$). For example, when quintiles of income were used as five SES groups (*k* = 5), the group with the lowest level of socioeconomic deprivation (highest income) *i* = 1 covered *PR*_1_ = [0%  − 20%], the second group (second-highest income) *i* = 2 covered *PR*_2_ = [20%  − 40%], etc. To convert *PR* to a point estimate for the level of socioeconomic deprivation, the midpoint between the lower and the upper limit of the *PR* of each SES group was used. In the example above, the highest income category (lowest level of socioeconomic deprivation) was coded as the 10^th^ percentile, the second income category as the 30^th^ percentile, and so forth. With this approach, a low percentile indicates a low level of socioeconomic deprivation (high level of SES), while the 100^th^ percentile indicates the highest level of socioeconomic deprivation.

### Quality assessment

Quality assessment was performed in line with the original systematic review [6] using the following criteria [19]: representativeness of the sample; measurement and definition of the independent and dependent variables; linkage of survey/census data; age-adjustment (for details of each criterion see Additional file 1: Table S5).

### Statistical analysis

The units of observation used for all analyses were the RRs relating to all pairwise comparisons reported in each study, where the level of socioeconomic deprivation has been coded in percentiles as described above. All models were fit to the level of socioeconomic deprivation, scaled as a proportion between 0 and 1.

To find out if there are substantial differences in the risk of dying from an alcohol-attributable cause of death between indicators of SES and sex once the aspect of “conventional groupings” for each indicator of SES (such as quintiles for income or three large categories for education), we performed three stepwise random-effects meta-regression models. We investigated the heterogeneity in the RR point estimates explained by (1) the level of socioeconomic deprivation (in continuous percentiles, as explained above), (2) the specific indicator of SES used (categorical variable), and (3) sex, while controlling for clustering of point estimates (pairwise comparisons) within studies (using a fixed-effect for study ID). A Hartung-Knapp-Sidik-Jonkman estimator was used for the random effects meta-regressions [20]. The covariates were consecutively introduced in the model. Model fit between different models was compared using ANOVA and R-squared measures. We used permutation tests to test the robustness of the model coefficients and avoid overfitting [21, 22]. Random-effects meta-regression models were calculated in R version 3.6.1 [23].

To investigate dose-response relationships, we first visually inspected the dose-response relationship between the level of socioeconomic deprivation and the logarithm of the relative alcohol-attributable mortality risk (all pairwise comparisons reported in a study), using bubble plots with locally weighted scatterplot smoothing (LOWESS) and simple inverse variance weights. Bubble plots were stratified by sex and indicator of SES (education, income, or occupation).

We then fitted one-stage random-effects dose-response meta-analyses using restricted maximum likelihood estimators to the level of socioeconomic deprivation and the logarithm of the RR of dying from an alcohol-attributable cause of death [24, 25]. Models were stratified by indicator of SES and sex. To investigate the shape of the dose-response relationships, we used polynomial transformations, adding stepwise a quadratic and a cubic term to the linear model. Model selection was based on statistical indicators (Akaike information criterion [AIC], R-squared, and statistical significance of the model predictors) and visual inspection of bubble plots. Dose-response analyses were performed in Stata 15 using the drmeta package [26].

Sensitivity analyses were carried out to investigate (1) the impact of study quality (i.e., all criteria fulfilled vs. at least one criterion not fulfilled); (2) any systematic differences between findings from Finland, which were over-represented in the sample, and other countries; and (3) the influence of including economic inequality (Gini coefficient) at the time of the baseline assessment in the meta-regression models.

## Results

**Fig. 1:**
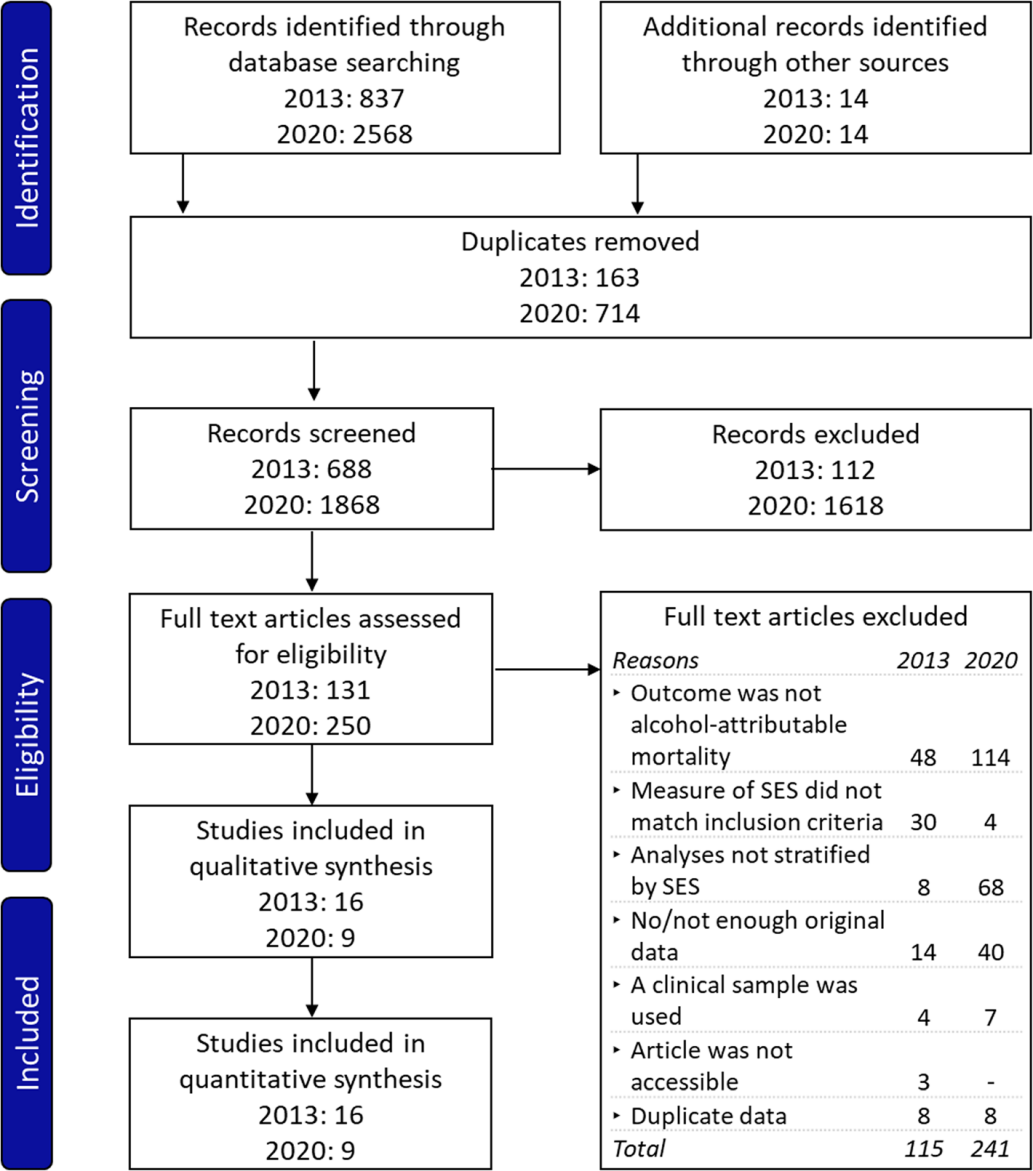
PRISMA flow chart of study selection for the search conducted in 2013 and 2020. SES, socioeconomic status

A total of 25 studies were included in the systematic review, 16 of which were retained from the review performed in 2013, and nine of which were newly identified (Fig. 1).

In total, the included studies reported findings based on about 241 million women and 230 million men, among whom there were about 75,200 and 308,400 alcohol-attributable deaths, respectively (Table 1). The review included data from 21 countries, with 11 studies reporting findings from Finland, followed by four each from Spain and the UK, and one to three for each of the remaining 18 countries. The studies reported on data spanning over 45 years from 1970 up until 2016. Education was the SES indicator most frequently studied (*n*=13 studies), followed by occupation (*n*=10) and income (*n*=4).

**Table 1:** Study characteristics of all studies included in the dose-response meta-analyses

Reference	Country, region/city	Study years	Age range (years)	Indicator of socioeconomic status (number of groups)	Sample size by sex	Number of deaths by sex	Study quality
Christensen et al. 2017 [27]	Denmark, Copenhagen, and Aarhus	1981–2009	30–70	Education (2)	38,982 (W), 35,287 (M)	87 (W), 270 (M)	(−)
Connolly et al., 2010 [28]	UK, Northern Ireland	2001–2006	25–74	Education (4), occupation (4)	369,245 (W), 351,382 (M)	201 (W), 377 (M)	(−)
Faeh et al., 2010 [29]	Switzerland	1990–2000	30–69	Education (3)	1,779,617 (W), 1,670,503 (M)	3911 (W), 12,245 (M)	(−)
Hemström, 2002 [30]	Sweden	1980–1995	20–64	Occupation (3)	749,260 (W), 730,789 (M)	1781 (W), 7766 (M)	(+)
Herttua et al. 2017 [31]	Finland	1988–2007	30–79	Education (3)	31,234,734 (W), 29,375,870 (M)	10,290 (W), 52,294 (M)	(−)
	Sweden	1991–2008	30–79	Education (3)	46,921,357 (W), 46,187,540 (M)	5653 (W), 23,038 (M)	
Herttua et al., 2008 [32]	Finland	2000–2003	30–80	Occupation (4)	2,018,000 (W), 1,891,000 (M)	555 (W), 2749 (M)	(+)
Herttua et al., 2011 [33]	Finland	2000–2007	15–79	Income (10)	226,930 (W), 219,890 (M)	1745 (W), 9770 (M)	(−)
Leinsalu et al., 2003 [34]	Estonia	1987–1990	20–70	Education (3)	610,006 (W), 495,219 (M)	83 (W), 334 (M)	(−)
Mäkelä et al., 1997 [35]	Finland	1985–1993	20–90	Occupation (3)	170,185 (W), 1,547,500 (M)	2809 (W), 18,026 (M)	(+)
Mäki et al., 2008 [36]	Finland	1990–2001	25–64	Income (4)	1,051,626 (M)	2703 (M)	(−)
Mäki et al., 2009 [37]	Finland	1990–2001	25–64	Income (4)	1,109,497 (W)	563 (W)	(−)
Mackenbach et al., 2015 [11]	Austria	2001–2002	35–79	Education (3), occupation (3)	2,210,000 (W), 2,038,000 (M)	102 (W), 403 (M)	(+)
	Belgium	2004–2005	35–79	Education (3)	5,561,000 (W), 5,251,000 (M)	644 (W), 1452 (M)	
	Czechia	1998–2003	35–79	Education (3)	3,268,000 (W), 2,929,000 (M)	1461 (W), 4982 (M)	
	Denmark	1991–2005	35–79	Education (3), occupation (3)	2,782,000 (W), 2,665,000 (M)	2848 (W), 7961 (M)	
	Estonia	1998–2002	35−79	Education (3), occupation (3)	499,400 (W), 379,700 (M)	774 (W), 2139 (M)	
	Finland	2006–2010	35–79	Education (3), occupation (3)	1,723,000 (W), 1,640,000 (M)	1950 (W), 7001 (M)	
	France	1990–2007	35–79	Education (3), occupation (3)	579,000 (W), 552,000 (M)	274 (W), 932 (M)	
	Hungary	1988–2002	35–79	Education (3)	6,141,500 (W), 5,158,000 (M)	9451 (W), 30,751 (M)	
	Italy, Turin	2006–2010	35–79	Education (3), occupation (3)	244,500 (W), 212,000 (M)	9 (W), 38 (M)	
	Lithuania	2001–2009	35–69	Education (3), occupation (3)	1,731,000 (W), 1,458,000 (M)	2073 (W), 5287 (M)	
	Norway	1990–2009	40–79	Education (3)	3,204,000 (W), 3,151,000 (M)	1526 (W), 4211 (M)	
	Poland	2001–2003	35–64	Education (3)	11,267,000 (W), 10,686,000 (M)	1038 (W), 7917 (M)	
	Slovenia	2002–2006	35–79	Education (3)	631,000 (W), 560,000 (M)	913 (W), 2820 (M)	
	Spain, Barcelona	1992–2010	35–79	Education (3)	3,879,000 (W), 3,504,000 (M)	348 (W), 1040 (M)	
	Spain, Madrid	1996–1997	35–79	Education (3)	605,000 (W), 522,000 (M)	5 (W), 26 (M)	
	Switzerland	1990–2008	35–79	Education (3), occupation (3)	5,209,000 (W), 4,543,000 (M)	2598 (W), 5442 (M)	
	UK, England and Wales	2006–2009	35–79	Education (3), occupation (3)	177,000 (W), 166,000 (M)	55 (W), 93 (M)	
	UK, Scotland	2006–2010	35–79	Education (3)	80,000 (W), 73,000 (M)	53 (W), 129 (M)	
	Sweden	1995–1999	35–64	Occupation (3)	1,967,000 (M)	2020 (M)	
Mackenbach et al., 2008 [10]	Belgium	1991–1995	30–74	Education (3)	2,805,780 (W), 2,718,890 (M)	2200 (W), 11,300 (M)	(+)
	Denmark	1996–2000	30–74	Education (3)	1,571,700 (W), 1,523,030 (M)	1200 (W), 6400 (M)	
	Italy, Turin	1991–2001	30–74	Education (3)	247,500 (W), 239,810 (M)	400 (W), 2200 (M)	
	Spain, Basque Country	1996–2001	30–74	Education (3)	1,860,466 (W), 1,802,867 (M)	300 (W), 1700 (M)	
Martikainen et al., 2001 [38]	Finland	1990–1995	35–85	Occupation (2)	1,170,200 (W), 976,400 (M)	723 (W), 3448 (M)	(−)
Mateo-Urdiales et al. 2020 [39]	Spain	2004–2011	≥35	Education (3)	25,050,004 (W), 22,601,192 (M)	1690 (W), 9684 (M)	(+)
Pechholdová and Jasilionis, 2020 [40]	Lithuania	2011–2016	≥30	Education (3)	1,340,504 (W), 1,028,114 (M)	936 (W), 2287 (M)	(+)
	Czechia	2011–2012	≥30	Education (3)	3,611,973 (W), 3,325,221 (M)	378 (W), 1037 (M)	
Pridemore et al., 2010 [41]	Russia, Izhevst	2003–2005	25–54	Education (6)	3149 (M)	100 (M)	(−)
Romeri et al., 2007 [42]	UK, England and Wales	2001–2005	20–64	Occupation (9)	17,504,000 (W), 17,217,000 (M)	3655 (W), 13,011 (M)	(−)
Shkolnikov et al., 1998 [43]	Russia	1989	20–69	Education (2)	46,306,000 (W), 43,130,000 (M)	2071 (W), 7139 (M)	(−)
Tarkiainen et al., 2016 [44]	Finland	1988–2012	35–64	Income (5)	242,500 (W), 244,400 (M)	4783 (W), 17,147 (M)	(+)
Tjepkema et al., 2013 [45]	Canada	1991–2006	25–64	Occupation (5)	1,153,500 (W), 1,082,400 (M)	490 (W), 1730 (M)	(+)
Tjepkema et al., 2012 [46]	Canada	1991–2006	25–80	Education (4)	1,376,600 (W), 1,358,200 (M)	1127 (W), 2990 (M)	(+)
Valkonen et al., 2000 [47]	Finland	1980–1985	35–64	Occupation (2)	741,200 (W), 681,200 (M)	650 (W), 3150 (M)	(+)
Valkonen, 1993 [48]	Finland	1970–1985	35–64	Occupation (4)	732,000 (M)	9000 (M)	(+)
Vierboom, 2020 [49]	USA	1990–2011	30–74	Education (4)	529,295 (W), 444,273 (M)	789 (W), 1823 (M)	(−)

*M* men, *W* women

(+) all quality criteria are met; (−) at least one quality criterion is not met

The majority of studies used a longitudinal design linking survey [27], census [10, 11, 28–30, 35–40, 45–48], or register data [31–33, 44] with cause-of-death registries (*n*=20). Four studies employed a cross-sectional design, using data from cause-of-death registries for mortality and census [34, 42, 43] or survey data [49] to inform population denominators to estimate mortality rates. One study employed a case-control design with cases taken from a death registry and controls sampled and frequency-matched from a population register [41]. For all 25 studies, sex-stratified estimates were available and for all but five studies [27, 29, 31, 34, 38] it was possible to extract age-adjusted estimates. None of the studies adjusted for another SES indicator.

### Study quality

About half of the studies (12 out of 25) fulfilled all quality criteria (see Additional file 1: Table S6). Of those that did not, five studies included causes of death that were not fully attributable to alcohol use, five studies did not use individual data linkage, six studies did not adjust for age (or did not allow for the calculation of age-adjusted results), and two studies did not satisfactorily measure SES (e.g., excluded meaningful parts of the population).

### Meta-regression

All three stepwise meta-regression models are shown in Additional file 1: Table S7. The final model, adjusting for the level of socioeconomic deprivation, SES indicator, and sex, explained 55% of the total heterogeneity in the RR point estimates. The level of socioeconomic deprivation was associated with a RR of 3.31 (95% CI 2.56–4.29), indicating an over three-fold higher alcohol-attributable mortality risk at the highest level of socioeconomic deprivation compared to the lowest level. In other words, assuming a linear relationship between the level of socioeconomic deprivation and the log RR, the risk of dying from an alcohol-attributable cause of death increased by about 13% with each decile increase in the level of socioeconomic deprivation.

Compared to occupation, education was associated with an RR of 1.87 (95% CI 1.45–2.42), indicating, on average, an 87% higher risk of dying when using education as a measure of SES as compared to occupation. Across all data points, men had a 16% higher risk of dying compared to women (RR 1.16, 95% CI 1.04–1.30). Permutation tests confirmed the findings from each of the three stepwise models (Additional file 1: Table S8).

### Dose-response models

**Fig. 2:**
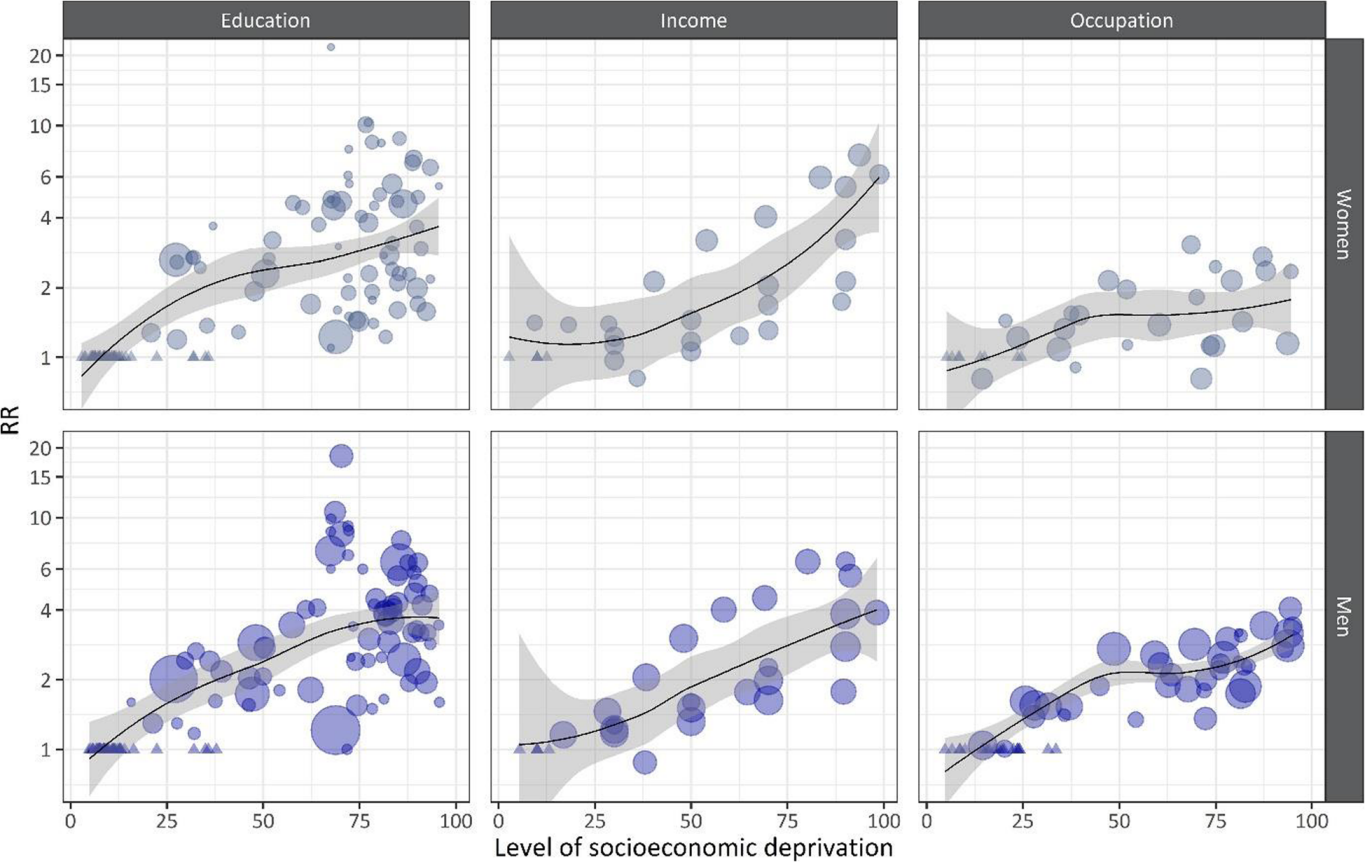
Bubble plots with locally weighted scatterplot smoothing for dose-response relationships between the level of socioeconomic deprivation and the relative alcohol-attributable mortality risk (RR, shown on the log scale) by indicator of socioeconomic status and sex (women in light blue, men in dark blue), using inverse variance weights. All pairwise comparisons reported in a study were included (unit of observation). The level of socioeconomic deprivation is coded as the midpoint of the percentile range in the cumulative SES distribution with 0=lowest level of socioeconomic deprivation and 100=highest level of socioeconomic deprivation. Triangles indicate reference groups, and bubbles show the RR point estimates according to their weight. The gray-shaded areas indicate the 95% confidence intervals around the locally weighted scatterplot smoothing lines

Bubble plots using LOWESS stratified by sex and indicator of SES show differences in the relationship between the level of socioeconomic deprivation and the RR concerning the shape of the relationship, as well as the RR associated with a high level of socioeconomic deprivation (Fig. 2).

The linear one-stage random-effects dose-response meta-analyses overall reflect the findings from the meta-regression. The addition of a quadratic term to the model considerably improved the predictive model fit, as indicated by the AIC, in most cases (detailed findings for linear models and models with quadratic term are shown in Additional file 1: Table S9). The addition of a cubic term did not considerably improve the fit for any of the SES indicator- and sex-specific models (results not shown). After visual inspection of the relationships and comparing all statistical indicators of fit, the most parsimonious model with the best fit was chosen (Table 2): In all but two cases, the model with the quadratic term was chosen, except for the dose-response relationship for income and occupation among men, where the linear model showed the best fit.

**Table 2:** Results from selected one-stage random-effects dose-response meta-analyses on the relative mortality risk for alcohol-attributable mortality conditional on the level of socioeconomic deprivation, stratified by sex and indicator of socioeconomic status (SES)

Model (N)	Women				Men			
Predictor	RR	95% CI	***R***^**2**^	AIC	RR	95% CI	***R***^**2**^	AIC
**Education**	**(*N*=71)**				**(*N*=76)**			
Level of deprivation	30.71***	9.96–94.67	0.27	125	50.71***	20.65–124.49	0.34	106
Level of deprivation^2^	0.15**	0.04–0.55			0.12***	0.05–0.33		
**Income**	**(*N*=24)**				**(*N*=24)**			
Level of deprivation	0.60	0.14–2.64	0.93	47	5.72***	3.43–9.80	0.81	976
Level of deprivation^2^	7.10**	2.06–24.51			-	-		
**Occupation**	**(*N*=24)**				**(*N*=37)**			
Level of deprivation	4.46***	2.38–8.35	0.32	225	4.16***	3.63–7.77	0.79	1367
Level of deprivation^2^	0.49**	0.28–0.86			-	-		

*N* number of risk estimates, *CI* confidence interval, *AIC* Akaike information criterion

*Note.* Models were fit to the level of socioeconomic deprivation, scaled as a proportion between 0 and 1. The coefficients for the level of socioeconomic deprivation refer to the difference between the lowest level of socioeconomic deprivation (i.e., lowest percentile) and the highest level of socioeconomic deprivation (100th percentile)

**Fig. 3:**
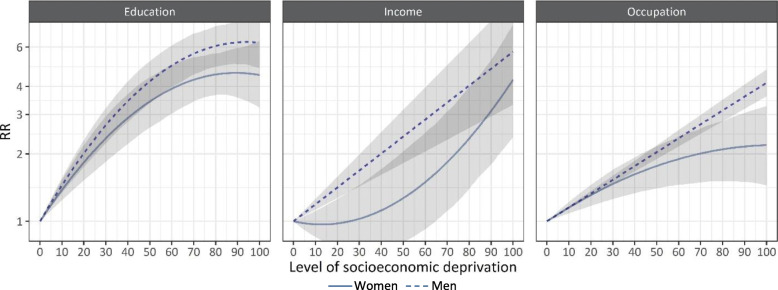
Dose-response relationship between the level of socioeconomic deprivation and the relative risk of mortality from an alcohol-attributable cause of death (RR, shown on the log scale) by the indicator of socioeconomic status (SES) and sex. The level of socioeconomic deprivation indicates the percentile in the cumulative SES distribution with 0=lowest level of socioeconomic deprivation and 100=highest level of socioeconomic deprivation. Gray-shaded areas show 95% uncertainty bands

Dose-response relationships based on the selected models are shown in Fig. 3 (respective dose-response relationships without log scale are shown in Additional file 1: Fig. S1). The sharpest increase in the RR of dying from an alcohol-attributable cause of death with increasing levels of socioeconomic deprivation was observed for education. At the 25^th^ percentile of the SES distribution, the RR was already above two among women (RR 2.09, 95% CI 1.70–2.59) and men (RR 2.34, 95% CI 1.98–2.76). This means that a level of education where only 24% of the population has a higher level of education is associated with a twofold risk of dying from an alcohol-attributable cause of death compared to individuals with the highest level of education. At the 60^th^ percentile, the RR of dying reaches 3.91 (women; 95% CI 3.10–5.04) and 4.97 (men; 95% CI 4.00–6.15) and does not increase considerably thereafter. In contrast, the alcohol-attributable mortality risk is twofold for individuals at the 40^th^ (men; RR 2.01, 95% CI 1.61–2.50) and the 75^th^ percentile (women, RR 2.07, 95% CI 1.21–3.58) of the income distribution, compared to individuals with the highest level of income. While for men the RR increases steadily as the level of income decreases, reaching a RR of nearly six at the lowest income level (100^th^ percentile: RR 5.72, 95% CI 3.30–9.88), for women the RR increases more sharply as the level of income decreases, from two at the 75^th^ percentile of the income distribution to a RR of about four for women with the lowest income level (100^th^ percentile: RR 4.29, 95% CI 2.50–7.40). The dose-response relationship for occupation shows the most pronounced sex differences at the lowest level of occupation (100^th^ percentile, women: RR 2.19, 1.48–3.23; 100^th^ percentile, men: RR 4.16, 95% CI 3.62–4.77). Sensitivity analyses did not indicate potential sources of systematic bias (Additional file 1: Table S10).

## Discussion

The present study is the most comprehensive overview of socioeconomic inequalities in alcohol-attributable mortality to date. We were able to show clear dose-response relationships for three indicators of SES for both sexes and found considerable differences in the shape of the dose-response relationship and the level of risk associated with socioeconomic deprivation. This study therefore strongly demonstrates the necessity to think about socioeconomic gradients rather than categories where those with a low SES are being perceived as the fringe of the society with elevated risks that are somehow different from the “general population”. Furthermore, the differences between the dose-response relationships point to distinct causal pathways connecting socioeconomic deprivation to increased mortality risks. These pathways need to be studied and accounted for rather than treating education, occupation, and income as equivalent indicators of a single latent variable of SES. This is in line with empirical [50] and theoretical work [12] on the interlinkages between different indicators of SES and their specific links to “distinct proximate determinants of health and mortality” (p. 555) [12]. As such, understanding the health benefits of all three indicators independently is integral to reducing health disparities, including but not limited to alcohol-attributable mortality.

With that in mind, the findings suggest that of the three indicators studied here, education is a particularly strong indicator of SES concerning impacts on alcohol-attributable mortality. Individuals with a medium or low level of education have a three- to fivefold higher alcohol-attributable mortality risk compared to individuals with high education. Interestingly, the alcohol-attributable mortality risk is similar for the bottom 20% to 30% of the education distribution. Strong gains in lowering the mortality risk can only be expected beyond this range. This may indicate some support of moving everybody beyond the threshold minimal education [51].

The RR for education is higher overall than the RR for occupation or income. This finding is surprising as a previous meta-analysis had found that compared to income, employment status, and occupation, education was associated with the lowest RR among men (2.88, 95% CI 2.45–3.40) and the second-lowest among women (RR 2.66, 95% CI 2.19–2.23) [9]. This previous meta-analysis was not able to account for the continuous level of socioeconomic deprivation but rather looked at extreme group comparisons, which are confounded with the type of SES indicator chosen. However, the current study also found a high level of heterogeneity and unexplained variability in the point estimates for education which warrant further study.

Relative mortality risks observed for occupation were overall lower. Interestingly, the results showed high sex differences in the relative mortality risks at the bottom end of the occupation spectrum (about twofold higher for men), confirming findings from previous research [9]. However, while the heterogeneity in the point estimates was comparatively low for men, the level of socioeconomic deprivation only explained a small proportion of the variability in the point estimates for women. This may be related to the historical practice of assessing the occupation of the head of the household (typically the husband) and applying it to all other individuals in the household, which may not accurately reflect the living situation of the female household member [12]. Furthermore, additional factors such as a lower income, child care, and societal standing play a stronger role in influencing risks for women above and beyond the occupation.

For income, the dose-response relationship showed that at the bottom end of the income distribution even small gains in income can go a long way in reducing the alcohol-attributable mortality risk, for women in particular. This has important implications for the social support and welfare system, indicating that moving individuals up the income distribution may have considerable preventive and public health effects.

### Limitations

One limitation of the current study is the assumption of a uniform distribution of SES within categories when calculating the level of socioeconomic deprivation. While using cumulative percentiles and the midpoint in each category as the level of socioeconomic deprivation is the best approach currently available, this approach does not account for the percentile range covered by each group. Especially in SES groups covering a broader percentile range, the mean level of socioeconomic deprivation may not have been estimated accurately as within each SES category individuals were not always uniformly distributed. With that being said, we would like to acknowledge that the term deprivation is typically used to capture more than simply varying levels of education, income, and occupation. In the context of this study, the term was used to indicate a decreasing level of SES on a continuous scale.

Furthermore, differences in the study design of the included studies may have introduced bias. Specifically, four studies used a cross-sectional design where data were not linked on the individual level. This can lead to the so-called “numerator-denominator bias” where the information on SES for the denominator is based on self-report in census or survey data, whereas the SES of the deceased (numerator) is assessed at the time of death via proxy informants which is generally considered to generate lower-quality data [48, 52, 53]. While there is some evidence for the bias leading to an underestimation of the socioeconomic inequalities [54], other studies found no systematic or the opposite effect [55, 56].

Only four studies, all of which came from Finland, used income as the SES indicator. However, we conducted a sensitivity analysis to appraise if overall results from Finland differed systematically from those in all other countries and did not find any evidence for such differences. Nevertheless, future studies are required from other countries to obtain generalizable evidence on the dose-response relationship between income levels and alcohol-attributable mortality risk.

It should be noted that while the target population was the general adult population, a bias may have been introduced when using occupation as an indicator of SES. This concerns (a) the issues related to assigning the occupation among women (discussed above) [12] and (b) excluding individuals outside the labor force which may again disproportionately affect women [57]. Individuals with an occupation may be healthier and differ in other relevant aspects from the total general adult population, potentially biasing the findings to show lower socioeconomic inequalities across the socioeconomic spectrum [58]. While all studies that considered occupation as an indicator of SES used a hierarchical approach, occupation tends to be much more sensitive to temporary fluctuations than education and income. Occupational profiles change over time, with traditional categories being superseded by new professions.

Given that people of low SES tend to experience greater alcohol-related harm than those of high SES, even when the amount of alcohol consumption is the same or less than for individuals of high SES (i.e., the so-called alcohol-harm paradox) [7, 8], it would have been ideal if we could have controlled for alcohol use in the included point estimates. However, this was not possible. Even though drinking patterns have been shown to explain only less than 30% of the socioeconomic inequalities in mortality [7], systematic differences in drinking patterns by SES and sex likely impact the shape of the sex-specific dose-response relationship. For example, women with a high level of education are about twice as likely to be current drinkers whereas for men those with high education are only 50% more likely to be current drinkers compared to men with low education [59].

## Conclusions

The findings of this study show that increased alcohol-attributable mortality is experienced across the entire spectrum of SES, with varying degrees of risk, which are the highest at the bottom of the spectrum. In line with previous research, the study reconfirms the importance of alcohol use as a contributing factor to socioeconomic inequalities in mortality [6, 60, 61]. This calls for increased research efforts into the SES-specific effects of alcohol control policies [62–64]. Specifically, policies that reduce the prevalence of heavy regular and heavy episodic drinking may be suitable to reduce socioeconomic inequalities as such drinking patterns have been shown to contribute to socioeconomic inequalities in mortality above and beyond the average level of consumption [7]. However, it has been found that differences in drinking patterns alone do not sufficiently explain the observed socioeconomic differences in alcohol-attributable mortality [7]. Thus, targeted alcohol control intervention strategies have to be supported by wider efforts to reduce inequalities in income, education, and the social, financial, and health hazards related to different occupations [65].

## Supplementary Information

**Additional file 1: **The dose-response relationship between socioeconomic deprivation and alcohol-attributable mortality risk – a systematic review and meta-analysis includes **Tables S1** to **S10**, **Text S1**, and **Fig. S1**. **Table S1** PRISMA 2009 checklist. **Text S1.** Search terms. **Table S2.** Inclusion criteria. **Table S3.** Diagnoses 100% attributable to alcohol use with ICD-10 codes. **Table S4.** Diagnoses with an alcohol-attributable fraction > 10% for mortality globally as per the Global Status Report on Alcohol and Health, 2018 (100% attributable causes are excluded from this table and shown in Table S3). **Table S5** Quality assessment criteria and ratings. **Table S6.** Quality checklist. Ratings on population representativeness of the sample, measurement of socioeconomic status (SES), operationalization of alcohol-attributable mortality, data linkage, and age-adjustment for each study included in the meta-analysis. **Table S7.** Results from random-effects meta-regression models predicting the relative risk (RR). **Table S8.** Results from permutation tests to test the robustness of random-effects meta-regression models predicting the log relative risk (RR) conditional on the level of socioeconomic deprivation, the indicator of socioeconomic status (SES) used, and sex in three consecutive models, while controlling for clustering of estimates within studies. **Table S9.** Results from one-stage random-effects dose-response meta-analyses on the relative mortality risk for alcohol-attributable mortality conditional on the level of socioeconomic deprivation, stratified by sex and indicator of socioeconomic status (SES). **Fig. S1.** Dose-response relationship between the level of socioeconomic deprivation and the relative risk of mortality from an alcohol-attributable cause of death (RR) by indicator of socioeconomic status (SES) and sex. **Table S10.** Results from sensitivity analyses. Random-effects meta-regression models predicting the log relative risk (RR) conditional on the study quality (a least one criterion not fulfilled vs. all criteria fulfilled), the country where the study was conducted (Finland vs. any other country), and the income inequality in the country (Gini coefficient).

## Data Availability

The datasets generated and/or analyzed during the current study, as well as all of the code, will be made available after publication in the figshare repository, doi: 10.6084/m9.figshare.13625576 [18].

## Abbreviations

AIC: Akaike information criterion
CI: Confidence interval
LOWESS: Locally weighted scatterplot smoothing
PRISMA: Preferred Reporting Items for Systematic Reviews and Meta-Analyses
RR: Relative risk
SES: Socioeconomic status
