# Supplementary material for: The dose-response relationship between socioeconomic deprivation and alcohol-attributable mortality risk—a systematic review and meta-analysis
Source: BMC Med. 2021 Nov 5;19:268. doi: 10.1186/s12916-021-02132-z (PMC8569998; doi:10.1186/s12916-021-02132-z)
Supplement: Supplementary file 1 — Additional file 1: The dose-response relationship between socioeconomic deprivation and alcohol-attributable mortality risk – a systematic review and meta-analysis includes Tables S1 to S10, Text S1, and Fig. S1. Table S1 PRISMA 2009 checklist. Text S1. Search terms. Table S2. Inclusion criteria. Table S3. Diagnoses 100% attributable to alcohol use with ICD-10 codes. Table S4. Diagnoses with an alcohol-attributable fraction > 10% for mortality globally as per the Global Status Report on Alcohol and Health, 2018 (100% attributable causes are excluded from this table and shown in Table S3). Table S5 Quality assessment criteria and ratings. Table S6. Quality checklist. Ratings on population representativeness of the sample, measurement of socioeconomic status (SES), operationalization of alcohol-attributable mortality, data linkage, and age-adjustment for each study included in the meta-analysis. Table S7. Results from random-effects meta-regression models predicting the relative risk (RR). Table S8. Results from permutation tests to test the robustness of random-effects meta-regression models predicting the log relative risk (RR) conditional on the level of socioeconomic deprivation, the indicator of socioeconomic status (SES) used, and sex in three consecutive models, while controlling for clustering of estimates within studies. Table S9. Results from one-stage random-effects dose-response meta-analyses on the relative mortality risk for alcohol-attributable mortality conditional on the level of socioeconomic deprivation, stratified by sex and indicator of socioeconomic status (SES). Fig. S1. Dose-response relationship between the level of socioeconomic deprivation and the relative risk of mortality from an alcohol-attributable cause of death (RR) by indicator of socioeconomic status (SES) and sex. Table S10. Results from sensitivity analyses. Random-effects meta-regression models predicting the log relative risk (RR) conditional on the study quality (a least one cri [file 12916_2021_2132_MOESM1_ESM.pdf]

Additional file 1 for

The dose-response relationship between socioeconomic deprivation and alcohol-attributable mortality risk – a systematic review and meta-analysis

Charlotte Probst, Shannon Lange, Carolin Kilian, Celine Saul, & Jürgen Rehm

Correspondence to: Charlotte Probst; Email: mariecharlotte.probst@gmail.com

This PDF file includes:

- Tables S1 to S10
- Text S1
- Fig. S1

PRISMA 2009 Checklist

Table S1 PRISMA 2009 checklist

Section/topic	#	Checklist item	Reported on page #
**TITLE**			
Title	1	Identify the report as a systematic review, meta-analysis, or both.	Title page
**ABSTRACT**			
Structured summary	2	Provide a structured summary including, as applicable: background; objectives; data sources; study eligibility criteria, participants, and interventions; study appraisal and synthesis methods; results; limitations; conclusions and implications of key findings; systematic review registration number.	2
**INTRODUCTION**			
Rationale	3	Describe the rationale for the review in the context of what is already known.	4-5
Objectives	4	Provide an explicit statement of questions being addressed with reference to participants, interventions, comparisons, outcomes, and study design (PICOS).	5-6
**METHODS**			
Protocol and registration	5	Indicate if a review protocol exists, if and where it can be accessed (e.g., Web address), and, if available, provide registration information including registration number.	6
Eligibility criteria	6	Specify study characteristics (e.g., PICOS, length of follow-up) and report characteristics (e.g., years considered, language, publication status) used as criteria for eligibility, giving rationale.	6, Table S2
Information sources	7	Describe all information sources (e.g., databases with dates of coverage, contact with study authors to identify additional studies) in the search and date last searched.	6
Search	8	Present full electronic search strategy for at least one database, including any limits used, such that it could be repeated.	6, see PROSPERO protocol for complete search terms
Study selection	9	State the process for selecting studies (i.e., screening, eligibility, included in systematic review, and, if applicable, included in the meta-analysis).	6
Data collection process	10	Describe method of data extraction from reports (e.g., piloted forms, independently, in duplicate) and any processes for obtaining and confirming data from investigators.	6
Data items	11	List and define all variables for which data were sought (e.g., PICOS, funding sources) and any assumptions and simplifications made.	7
Risk of bias in individual studies	12	Describe methods used for assessing risk of bias of individual studies (including specification of whether this was done at the study or outcome level), and how this information is to be used in any data synthesis.	7, Table S3

Section/topic	#	Checklist item	Reported on page #
Summary measures	13	State the principal summary measures (e.g., risk ratio, difference in means).	7-8
Risk of bias across studies	15	Specify any assessment of risk of bias that may affect the cumulative evidence (e.g., publication bias, selective reporting within studies).	6
Synthesis of results	14	Describe the methods of handling data and combining results of studies, if done, including measures of consistency (e.g., I^2) for each meta-analysis.	7
Additional analyses	16	Describe methods of additional analyses (e.g., sensitivity or subgroup analyses, meta-regression), if done, indicating which were pre-specified.	8
**RESULTS**			
Study selection	17	Give numbers of studies screened, assessed for eligibility, and included in the review, with reasons for exclusions at each stage, ideally with a flow diagram.	10-11 Fig. 1
Study characteristics	18	For each study, present characteristics for which data were extracted (e.g., study size, PICOS, follow-up period) and provide the citations.	Table 1
Risk of bias within studies	19	Present data on risk of bias of each study and, if available, any outcome level assessment (see item 12).	Table S3, 6-7
Results of individual studies	20	For all outcomes considered (benefits or harms), present, for each study: (a) simple summary data for each intervention group (b) effect estimates and confidence intervals, ideally with a forest plot.	Table 1 as applicable
Synthesis of results	21	Present results of each meta-analysis done, including confidence intervals and measures of consistency.	11-13, Table 2, 3
Risk of bias across studies	22	Present results of any assessment of risk of bias across studies (see Item 15).	Table S4, 7
Additional analysis	23	Give results of additional analyses, if done (e.g., sensitivity or subgroup analyses, meta-regression [see Item 16]).	Table S5, 14; Fig. S1, 2
**DISCUSSION**			
Summary of evidence	24	Summarize the main findings including the strength of evidence for each main outcome; consider their relevance to key groups (e.g., healthcare providers, users, and policy makers).	14-15
Limitations	25	Discuss limitations at study and outcome level (e.g., risk of bias), and at review-level (e.g., incomplete retrieval of identified research, reporting bias).	16-17

Section/topic	#	Checklist item	Reported on page #
Conclusions	26	Provide a general interpretation of the results in the context of other evidence, and implications for future research.	17-18
**FUNDING**			
Funding	27	Describe sources of funding for the systematic review and other support (e.g., supply of data); role of funders for the systematic review.	19

From: Moher D, Liberati A, Tetzlaff J, Altman DG, The PRISMA Group (2009). Preferred Reporting Items for Systematic Reviews and Meta-Analyses: The PRISMA Statement. PLoS Med 6(7): e1000097. doi:10.1371/journal.pmed1000097

For more information, visit: www.prisma-statement.org.

Page 2 of 2

Text S1 Search terms

Databases

Systematic literature searches was performed in Embase, Medline and Psychinfo (via OvidSP) as well as Web of Science. The searches will include a set of keywords, wildcards, truncation and medical subject headings (where applicable). The search terms are shown below.

OvidSP

(alcohol *abuse/ OR (alcohol abuse).ti,ab. OR exp *Drinking Behavior/ OR exp *Alcohol Drinking/ OR exp *Drinking/ OR *Binge Drinking/ OR (alcoholic beverages).ti,ab. OR (alcohol and (drinking or intake or consumption)).ti,ab.)

AND

(exp *socioeconomic factors/ OR exp *social class/ OR socio?economic status.ti,ab. OR Educational status/ OR income/ OR Employment/ OR (education* ADJ (level OR attain*)).ti,ab. OR (socio?economic OR SES OR SEP OR asset? score OR income).ti,ab.)

AND

(exp Mortality/ OR exp Mortality, Premature/ OR exp *excess mortality/ OR *differential mortality/ OR (mortality).ti,ab. OR *cause of death/)

AND

(exp Case?Control Studies/ OR exp Cohort studies/ OR (case OR cohort OR control group* OR ratio OR risk* OR prospective* OR follow* OR longitudinal OR retrospective OR effect modifi*).ti,ab.)

Limit 1 to yr="2013-Current"

Web of Science

TS=(alcoholic beverages OR alcohol AND (drinking OR intake OR consumption)) OR TS=(Alcohol abuse)

AND

TS=(mortality OR death* OR cause of death)

AND

TS=(ses OR socioe$conomic status OR social class OR socio$economic variable* OR (education* AND (attain* OR status OR level)) OR income OR employment)

AND

TS=(case?control stud* OR cohort?stud* OR ratio OR risk* OR prospective* OR follow* OR longitudinal OR retrospective OR effect modifi*)

Table S2 Inclusion criteria

Criterion	Inclusion	Exclusion
Outcome	The outcome is mortality measured at the individual level	Indirectly affected people are investigated (e.g., sober victims of car drivers under the influence of alcohol)
	The outcome is attributable to alcohol use	
SES	SES is measured via occupation, income, or education	SES is measured on only one level (i.e., no comparison group)
	SES is measured at the individual, parental, or household level	SES is measured on the neighborhood-level
Design	The study is empirical and quantitative	The study is an intervention study
	The study employed a longitudinal design with data-linkage, a cross-sectional design (deaths with a population denominator), or a case-control design	Intervention studies
Sample	The sample is population-based	A clinical sample is investigated
	Participants are at least 15 years of age	
Results	The outcome (including N) is reported by SES of the deceased	
	One point estimate measure of relative risk (RR, odds ratio (OR), hazard ratio (HR), standardized mortality ratio (SMR), etc.) comparing risk in different SES strata and its confidence interval, standard error, or sufficient raw data for calculation (N total and n cases) are reported	
Language	No restrictions	
Time	Studies published since February 2013	

SES socioeconomic status.

Table S3 Diagnoses 100% attributable to alcohol use with ICD-10 codes

Diagnosis	ICD-10 code
Alcohol-induced pseudo-Cushing's syndrome	E24.4
Mental and behavioural disorders due to use of alcohol	F10
Acute intoxication	F10.0
Harmful use	F10.1
Dependence syndrome	F10.2
Withdrawal state	F10.3
Withdrawal state with delirium	F10.4
Psychotic disorder	F10.5
Amnesic syndrome	F10.6
Residual and late-onset psychotic disorder	F10.7
Other mental and behavioural disorders	F10.8
Unspecified mental and behavioural disorder	F10.9
Degeneration of nervous system due to alcohol	G31.2
Alcoholic polyneuropathy	G62.1
Alcoholic myopathy	G72.1
Alcoholic cardiomyopathy	I42.6
Alcoholic gastritis	K29.2
Alcoholic liver disease	K70
Alcoholic fatty liver	K70.0
Alcoholic hepatitis	K70.1
Alcoholic fibrosis and sclerosis of liver	K70.2
Alcoholic cirrhosis of liver	K70.3
Alcoholic hepatic failure	K70.4
Alcoholic liver disease, unspecified	K70.9
Alcohol-induced pancreatitis	K85.2
Alcohol-induced chronic pancreatitis	K86.0
Finding of alcohol in blood	R78.0
Toxic effect of alcohol	T51
Toxic effect of ethanol	T51.0
Toxic effect of methanol	T51.1
Toxic effect of other alcohols	T51.8
Toxic effect of alcohol, unspecified	T51.9
Accidental poisoning by and exposure to alcohol	X45
Intentional self-poisoning by and exposure to alcohol	X65
Poisoning by and exposure to alcohol, undetermined intent	Y15
Evidence of alcohol involvement determined by blood alcohol level	Y90

Table S4 Diagnoses with an alcohol-attributable fraction >10% for mortality globally as per the Global Status Report on Alcohol and Health, 2018 (100% attributable causes are excluded from this table and shown in Table S3) [16]

Category	Sub-category	Cause of death	ICD-10 code	AAF
Communicable diseases	Respiratory infections and tuberculosis	Tuberculosis	A15-A19, B90	20%
Injuries	Intentional injuries	Self-harm	X60-64, X66-X69, X65, X70-X84	18%
		Interpersonal violence	X85–Y09, Y871	18%
	Unintentional injuries	Road injury	V01–V04, V06, V09–V80, V87, V89, V99	27%
		Poisonings	X40, X43, X46–X48, X49	12%
		Falls	W00–W19	11%
		Drowning	W65–W74	12%
		Exposure to mechanical forces	W20–W38, W40–W43, W45, W46, W49–W52, W75, W76	14%
		Other unintentional injuries	V2*, W39, W44, W53-W64, W77-W99, X20-X29, X50-X59, Y40-Y86, Y88, Y89	14%
Noncommunicable diseases	Digestive diseases	Cirrhosis of the liver	K70, K74	48%
		Pancreatitis	K85–K86	26%
	Malignant neoplasms	Lip and oral cavity	C00-C08	26%
		Other pharyngeal cancers	C09–C10, C12–C14	31%
		Oesophagus cancer	C15	17%
		Colon and rectum cancers	C18-C21	11%
		Larynx cancer	C32	22%
	Neurological disorders	Epilepsy	G40-G41	13%

* V-series not included in road injuries
AAF alcohol-attributable fraction.

Table S5 Quality assessment criteria and ratings

Criterion	Categories	Evaluation	Code
Sample representativeness	Nationally representative sample	Satisfactory	0
	Fraction of the target population	Insufficient	1
Assessment of SES	Assigned based on most recent census	Satisfactory	0a
	Assigned through family or household member	Satisfactory	0b
	Use of an own or 'other' category	Satisfactory	0c
	One or more pairwise comparisons are not reported	Satisfactory	0d
	Exclusion of meaningful parts of the population	Insufficient	1
Definition of alcohol-attributable mortality	100% alcohol-attributable mortality exclusively	Satisfactory	0
	Inclusion of diseases of the liver and/or pancreatitis	Insufficient	1a
	Inclusion of neoplasms in the upper gastrointestinal tract	Insufficient	1b
	Inclusion of all types of injures	Insufficient	1c
	Inclusion of two or more of the above categories	Flawed	2
Data linkage	Individual linkage	Satisfactory	0
	No linkage	Insufficient	1
Age adjustment	Age-adjusted	Satisfactory	0
	Not age-adjusted	Insufficient	1

SES socioeconomic status.

Table S6 Quality checklist. Ratings on population representativeness of the sample, measurement of socioeconomic status (SES), operationalization of alcohol-attributable mortality, data linkage, and age-adjustment for each study included in the meta-analysis

Study	Population representa-tiveness of the sample	Measure-ment of SES	Operationalization of alcohol-attributable mortality	Data linkage	Age adjustment
Valkonen, 1993 [48]	0	0a	0	0	0
Mäkelä et al., 1997 [35]	0	0b	0	0	0
Shkolnikov et al., 1998 [43]	0	0	0	1	0
Valkonen et al., 2000 [47]	0	0a,b	0	0	0
Martikainen, 2001 [38]	0	0a	0	0	1
Hemström, 2002 [30]	0	0a,c	0	0	0
Leinsalu et al., 2003 [34]	0	0	0	1	0
Romeri et al., 2007 [42]	0	0	0	1	0
Herttua et al., 2008 [32]	0	0a,b	0	0	0
Mackenbach et al., 2008 [10]	0	0	0	0	0
Mäki et al., 2008 [36]	0	1	1c	0	0
Mäki et al., 2009 [37]	0	1	1c	0	0
Conolly et al., 2010 [28]	0	0	1a	0	0
Faeh et al., 2010 [29]	0	0	2	0	1
Pridemore et al., 2010 [41]	0	0	0	1	0
Herttua et al., 2011 [33]	0	0a,b	0	0	1
Tjepkema et al., 2012 [46]	0	0	0	0	0
Tjepkema et al., 2013 [45]	0	0	0	0	0
Mackenbach et al., 2015 [11]	0	0d	0	0	0
Tarkiainen et al., 2016 [44]	0	0b	0	0	0
Christensen et al., 2017 [27]	0	0	0	0	1
Herttua et a., 2017 [31]	0	0a,b	0	0	1
Mateo-Urdiales et al., 2020 [39]	0	0	0	0	0
Pechholdová & Jasilionis 2020 [40]	0	0	0	0	0
Vierboom, 2020 [49]	0	0	2	1	0

0=Satisfactory, criterion is met (e.g., sample is representative; data were linked etc.)
0a=Classification via a previous census
0b=Classification via another family member
0c=Formation of a rest category
0d=One or more pairwise comparisons are not reported

1=Not satisfactory, criterion is not met
1a=Diseases of liver and/or pancreas are included
1b=Neoplasms of the upper gastrointestinal tract are included
1c=Injuries of all kind are included
1d=The age ranged spanned less than 10 years
2=More than one of the disease categories mentioned above are included

Table S7 Results from random-effects meta-regression models predicting the relative risk (RR)

Predictor[†]	RR	95% CI	R^2	AIC	ANOVA
**Model 1**					
Level of deprivation	3.33***	2.53-4.37	0.50	329	
**Model 2**					
Level of deprivation	3.34***	2.57-4.34	0.54	308	***
Education	1.77***	1.38-2.29			
Income	1.15	0.76-1.75			
**Model 3**					
Level of deprivation	3.31***	2.56-4.29	0.55	301	**
Education	1.87***	1.45-2.42			
Income	1.23	0.81-1.86			
Male sex	1.16**	1.04-1.30			

CI confidence interval; AIC Akaike information criterion.

* p<0.1, ** p<0.05, ***p<0.001.

[†] Reference groups used were occupation for the indicator of socioeconomic status (SES), and women for sex. Models were fit to the level of socioeconomic deprivation, scaled as a proportion between 0 and 1. The coefficients for the level of socioeconomic deprivation refer to the difference between the lowest level of socioeconomic deprivation (i.e., lowest percentile) and the highest level of socioeconomic deprivation (100th percentile).

Note. The level of socioeconomic deprivation, the indicator of SES used, and sex were introduced as covariates in three stepwise models. Fixed effects for the study ID were used to control for clustering of observations within studies.

Table S8 Results from permutation tests to test the robustness of random-effects meta-regression models predicting the log relative risk (RR) conditional on the level of socioeconomic deprivation, the indicator of socioeconomic status (SES) used, and sex in three consecutive models, while controlling for clustering of estimates within studies

**Predictor†**	**RR**	**p-value**	**95% CI**
**Model 1**			
Level of deprivation	3.33	0.001	2.53-4.37
**Model 2**			
Level of deprivation	3.34	0.001	2.57-4.34
Education	1.77	0.001	1.38-2.29
Income	1.15	0.483	0.76-1.75
**Model 3**			
Level of deprivation	3.31	0.001	2.56-4.29
Education	1.87	0.001	1.45-2.42
Income	1.23	0.349	0.81-1.86
Male sex	1.16	0.008	1.04-1.30

CI confidence interval.

† Reference groups used were occupation for the indicator of SES, and women for sex. The coefficients for the level of socioeconomic deprivation refer to the difference between the lowest level of deprivation (i.e., lowest percentile) and the highest level of socioeconomic deprivation (100th percentile).

Note. Fixed effects for the study ID were used to control for clustering of observations within studies.

Table S9 Results from one-stage random-effects dose-response meta-analyses on the relative mortality risk for alcohol-attributable mortality conditional on the level of socioeconomic deprivation, stratified by sex and indicator of socioeconomic status (SES)

**Model (N)**	**Women**				**Men**			
**Predictor**	**RR**	**95% CI**	**R^2**	**AIC**	**RR**	**95% CI**	**R^2**	**AIC**
**Education**	**(N=71)**				**(N=76)**			
Model 1								
Level of deprivation	5.16***	3.93-6.78	0.27	1481	7.28***	5.65-9.39	0.34	3358
Model 2								
Level of deprivation	30.71***	9.96-94.67	0.27	125	50.71***	20.65-124.49	0.34	106
Level of deprivation^2	0.15**	0.04-0.55			0.12***	0.05-0.33		
**Income**	**(N=24)**				**(N=24)**			
Model 1								
Level of deprivation	4.99***	2.76-9.02	0.92	190	5.72***	3.43-9.80	0.81	976
Model 2								
Level of deprivation	0.60	0.14-2.64	0.93	47	1.67	0.27-10.39	0.81	506
Level of deprivation^2	7.10**	2.06-24.51			3.03	0.50-18.41		
**Occupation**	**(N=24)**				**(N=37)**			
Model 1								
Level of deprivation	2.13***	1.44-3.16	0.30	242	4.16***	3.63-7.77	0.79	1367
Model 2								
Level of deprivation	4.46***	2.38-8.35	0.32	225	4.32**	1.80-10.35	0.85	691
Level of deprivation^2	0.49**	0.28-0.86			0.99	0.39-2.52		

N number of risk estimates; CI confidence interval; AIC Akaike information criterion.

Note. Models were fit to the level of socioeconomic deprivation, scaled as a proportion between 0 and 1. The coefficients for the level of socioeconomic deprivation refer to the difference between the lowest level of socioeconomic deprivation (i.e., lowest percentile) and the highest level of socioeconomic deprivation (100th percentile).

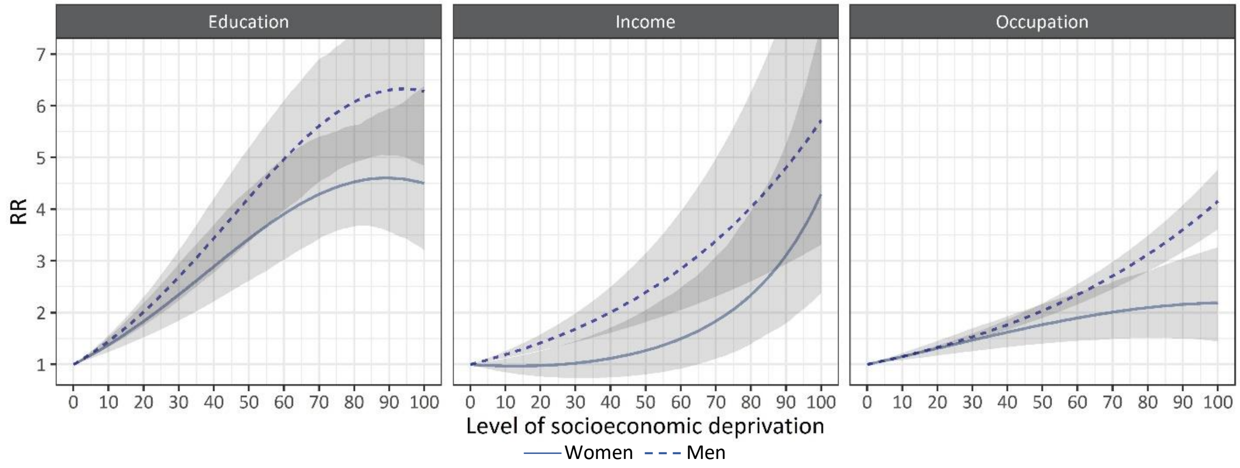

Fig. S1 Dose-response relationship between the level of socioeconomic deprivation and the relative risk of mortality from an alcohol-attributable cause of death (RR) by indicator of socioeconomic status (SES) and sex.

The level of socioeconomic deprivation indicates the percentile in the cumulative SES distribution with 0=lowest level of socioeconomic deprivation and 100=highest level of socioeconomic deprivation. Grey shaded areas show 95% uncertainty bands.

Table S10 Results from sensitivity analyses. Random-effects meta-regression models predicting the log relative risk (RR) conditional on the study quality (a least one criterion not fulfilled vs. all criteria fulfilled), the country where the study was conducted (Finland vs. any other country), and the income inequality in the country (Gini coefficient)

Predictor†	RR	p-value	95% CI
**Model 1**			
Study quality	0.93	0.747	0.62-1.41
**Model 2**			
Finland (vs. all other)	0.73	0.086	0.50-1.05
**Model 3**			
Gini coefficient	1.01	0.086	0.98-1.04

All models adjust for the level of socioeconomic deprivation. Fixed effects for the study ID were used to control for clustering of observations within studies.

CI confidence interval.
